# An observational study of the direct costs related to hospital admissions, mortality and premature death associated with liver disease in Portugal

**DOI:** 10.1186/s13104-016-1879-8

**Published:** 2016-02-03

**Authors:** Sofia Vitor, Rui Tato Marinho, José Gíria, José Velosa

**Affiliations:** Department of Gastrenterology and Hepatology, Hospital de Santa Maria, Centro Hospitalar Lisboa Norte, Avenida Professor Egas Moniz, 1649-035 Lisbon, Portugal; Direcção Geral de Saúde, Alameda D. Afonso Henriques,45, 1049-005 Lisbon, Portugal

**Keywords:** Portugal, Liver disease, Premature death, Direct costs, Mortality, I110, I140, I180, H510

## Abstract

**Background:**

Liver disease, one of the most common causes of hospitalization worldwide, is particularly prevalent in Europe. This study aimed to determine the number of hospital discharges and admissions, mortality, premature death and costs associated with liver disease from the perspective of the National Health Service in Portugal.

**Methods:**

A descriptive, retrospective analysis of data from 97 hospitals between 2000 and 2008, and mortality data for 2011 collected from the Portuguese National Institute of Statistics. The 9th and 10th revisions of the international classification of diseases were used to establish diagnoses. National data on demographics, average length of stay, in-patient mortality and direct costs associated with hospital admissions and liver transplantation were compared for the most common liver diseases. Mortality and premature death were compared using the potential years of life lost (PYLL) index.

**Results:**

The annual mean number of discharges for liver disease was 11,503 between 2000 and 2008. Most cases of liver disease were diagnosed in men (70.4 %) and the prevalence of liver disease peaked in patients aged from 20 to 64 years (60.7 %). Alcoholic cirrhosis was the most frequent liver-disease diagnosis leading to discharge (38.6 %). In addition, alcoholic cirrhosis emerged as the main cost-driver, accounting for €26,818,930 (42.6 %) of the total cost imposed by liver disease. Overall, chronic hepatic disease was the 10th most common cause of mortality in Portugal in 2011, causing 21.8 deaths per 100,000. Chronic hepatic disease and hepatocellular carcinoma are even more important causes of premature death, ranking third based on PYLL.

**Conclusion:**

In 2011, liver disease was the 10th most common cause of death and the third most important cause of premature death in Portugal. Alcohol cirrhosis was the leading cause of liver-related hospital admissions between 2001 and 2008. It appears that liver disease imposes a considerable social and economic burden on Portugal. Our results suggest that educational, legislative and therapeutic interventions to prevent morbidity, mortality and premature death from liver disease are urgently required to minimise the economic and clinical burdens.

## Background

Liver disease, one of the most common causes of hospitalization worldwide, [1, 2] is particularly prevalent in Europe [3]. Indeed, liver disease was the seventh most common cause of death in Europe during 2010 [4]. Chronic hepatitis, cirrhosis and hepatocellular carcinoma (HCC) are the most common liver diseases in Europe [3, 5]. Viruses and alcohol account for more than 75 % of cases of liver disease in Portugal [6]. For example, cirrhosis frequently causes morbidity and mortality [7–9], and the direct and indirect financial costs associated with liver diseases impose a considerable socioeconomic burden on health services and society [10].

Against this background, economic evaluations from the perspective of the Portuguese National Health Service inform policies to promote prevention and management of liver disease and other health problems [1, 2, 11–14]. However, although health professionals recognize liver diseases as a common cause of morbidity and mortality in Portugal [7, 9], few studies estimate the socioeconomic impact. Therefore, our study aims to determine the profile of patients admitted with liver disease between 2001 and 2008, including details of the average length of stay (ALOS), mortality among inpatients, and direct costs specifically associated with hospital admissions due to liver diseases to the National Health Service of Portugal. In addition, to place these results in context, we compared mortality and potential years of life lost (PYLL) associated with liver disease to those associated with other common conditions. This included all national deaths to common conditions, occurring both inside and outside of hospital, in 2011.

## Methods

### Population

This descriptive, retrospective analysis uses freely available admission and discharge data already in the public domain, which was obtained from the Portuguese Directorate General for Health or the ‘Direcção Geral de Saude’ (DGS). This included data from 97 public hospitals in the Portuguese National Health Service categorized using diagnosis related-groups (AP21 version) between 2000 and 2008. This timeframe was decided based on the available data as the time of data analysis. Patient variables (sex, age, mortality and ALOS) were analyzed using Excel 2003. All patients admitted by liver disease from 2000 to 2008 were included and their variables (sex, age, mortality and ALOS) were analyzed using Excel 2003. To identify the group of inpatients that included liver diseases, ICD-codes were used as a filter.

In addition, demographic data and mortality from 2011 were collected from another source, the Portuguese National Institute of Statistics [Instituto Nacional de Estatística (INE)] website (http://www.ine.pt) and analyzed using Excel 2003. This allowed us to quantify the mortality associated with liver disease and compare it with the mortality associated with other common causes of death in Portugal.

### Diagnoses of admissions and discharges

Data was obtained from the DGS for the most frequent causes of liver diseases (2000–2008) (Table 1), which were established using the international classification of diseases (ICD) 9th revision, clinical modification, (ICD‐9‐CM). Using ICD-10 (2008 version), we determined the disease categories that caused more deaths than liver disease in 2011 (Table 2).

**Table 1:** Categories of liver diseases

Disease category	ICD 9 CM Code
Viral hepatitis	070
HBV	070.3–070.4
HCV	070.41, 070.44, 070.51, 070.54, 070.7
Cirrhosis	571
Alcoholic cirrhosis	571.2
Non-alcoholic cirrhosis	571.5
HCC	155.0
Other^a^	570–573, excluding codes above

^a^Includes: hepatoptosis; alcoholic liver damage unspecified; autoimmune hepatitis; toxic hepatitis: hepatitis unspecified

**Table 2:** Categories of diseases with greater mortality than liver diseases in 2011

Disease category	Disease subcategory	ICD-10 Code
Cerebrovascular disease		I60–I69
Chronic hepatic disease and cirrhosis		K70–K77, B15–B19
Diabetes		E10–E14
HCC		C22.0
HIV		B20–B24
Ischemic heart disease		I20–I25
Malignant neoplasm		C18–C20
	Female breast	C50
	Prostate	C61
	Stomach	C16
	Bronchus and lung	C33–C34
Motor vehicle traffic accidents		V01–V89
Pneumonia		J09–J18

### Direct costs

We calculated the direct costs paid by Portuguese National Health Service for in-patients admitted to all 97 public hospitals with liver diseases between January 2000 and December 2008. Private institutions do not provide data to the DGS, nor is this data in the public domain. We also collected ALOS and the amount paid per day and for each admission, including medication, examinations, medical and nursing support, from hospital databases (all included in information obtained from the DGS). Therefore, this analysis includes the costs associated with inpatient stay only (e.g., drug costs, nursing, consultation and invasive treatment). Ambulatory costs were not available and any outpatient costs have not been captured. The total costs of inpatient care were produced as a function of ALOS and the amount paid per day per patient. No adjustment for cost was made depending on the reference year.

The authors of this study feel it is important to capture the costs related to liver transplantation as it is the only treatment that solves end-stage liver disease. Costs related to liver transplantation were collected and analyzed separately to the inpatient costs of liver disease outlined above. Some other treatments, such as albumin dialysis, are used as a bridge to liver transplantation and are often performed during the same admission.

### Premature death

Lost earnings due to morbidity and premature mortality is one of the most important drivers of indirect costs [15]. Therefore, we collected mortality data for people aged 70 years of age and younger, including stratification by cause and sex, from INE registries for 2011. Diseases considered were classified using ICD-10 codes. Based on this, we estimated the PYLL, a summary measure of premature mortality which provides an explicit way of weighting deaths occurring at younger ages, which are, a priori, preventable. The calculation of PYLL involves summarising deaths occurring at each age and multiplying this with the number of remaining years to live up to a selected age limit. In order to assure cross-country comparisons when calculating PYLL, a limit of 70 years is used [16]. This is an established method to describe specific patterns in premature mortality and a mechanism for establishing healthcare priorities [17]. In this study, PYLL values were calculated for people aged 70 years old and under, and those occurring due to liver disease were compared to those due to other conditions.

## Results

### Sample

The mean number of hospital discharges for liver disease was 11,503 annually. The number of discharges attributable to liver disease decreased slightly between 2000 (11,617) and 2008 (10,503) (Table 3). Cirrhosis remained the main cause of admission for liver disease between 2000 and 2008. Over the same time, discharges due to viral hepatitis almost halved (1509 and 674) while discharges for HCC doubled (684 and 1127).

**Table 3:** Number of discharges diagnosed with liver disease between 2000 and 2008

Year	Number diagnosed with liver disease				
	Viral hepatitis	Cirrhosis	HCC	Other^a^	All
2000	1509	5313	684	4111	11,617
2001	1317	5821	689	3755	11,582
2002	1744	5995	743	4067	12,549
2003	1599	5770	774	4330	12,473
2004	1399	5637	862	3963	11,861
2005	1087	5530	834	3997	11,448
2006	811	5431	862	3766	10,869
2007	811	5151	912	3752	10,627
2008	674	4769	1127	3933	10,503

^a^Includes: hepatoptosis; alcoholic liver damage unspecified; autoimmune hepatitis; toxic hepatitis; hepatitis unspecified

**Fig. 1:**
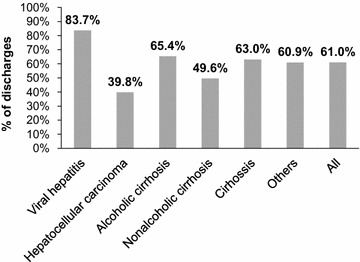
Proportion (%) of discharges among people aged 20–64 years in 2008 (n = 10,503)

Table 4 summarizes the number of discharges stratified by liver disease. Cirrhosis, mainly caused by alcohol abuse, was the most frequent indication leading to discharge (38.6 %), corresponding to 58.2 cases per 100,000 inhabitants. HCC was the second most common cause of hospital discharge, corresponding to 40 cases per 100,000 inhabitants. In general, males are more likely to be discharged with a liver disease than women (70.4 versus 29.6 %; overall discharge ratio: 2.4:1), with the greatest sex differences in HCC (3.8:1) and alcoholic cirrhosis (3.5:1). In 2008, the proportion of discharged patients aged from 20 to 64 years old was highest for hepatitis C virus (HCV) (86.9 %), followed by viral hepatitis generally (Fig. 1). Overall, this age group accounted for 60.7 % of patients discharged with liver disease in 2008.

**Table 4:** Number of discharges by liver disease categories and sex in 2008

Disease category	Disease subcategory	Number of discharges	% of all discharges	Discharge ratio male:female
Viral hepatitis	All	674	6.4	2.0
	HBV	210	2.0	2.1
	HCV	386	3.7	2.4
Cirrhosis	All	4769	45.4	3.0
	Alcoholic cirrhosis	4053	38.6	3.5
	Non-alcoholic cirrhosis	716	6.8	1.4
HCC		1127	10.7	3.8
Other^a^		3933	37.4	1.7
All		10,503	100	2.4

^a^Includes: hepatoptosis; alcoholic liver damage unspecified; autoimmune hepatitis; toxic hepatitis; hepatitis unspecified

### ALOS and mortality

**Fig. 2:**
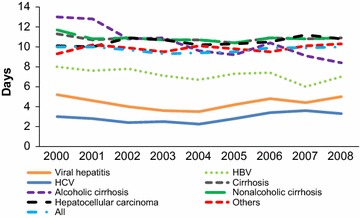
Average length of stay for liver diseases discharges (2000–2008) (*HBV* hepatitis B, *HCV* hepatitis C virus)

The ALOS for liver disease discharges overall and stratified by condition in 2008 was approximately 10 days, which showed little variation from 2000 to 2008. However, Fig. 2 shows that the ALOS for alcoholic cirrhosis decreased from 13 to 8 days during this time. In 2008, HCC and non-alcoholic cirrhosis were associated with the highest ALOS. Viral hepatitis had the shortest ALOS.

Table 5 shows mortality rates between 2000 and 2008. In 2008, HCC emerged as having the highest mortality rate among liver diseases (22.6 %), followed by alcoholic (14.9 %) and non-alcoholic cirrhosis (13.4 %). Mortality rate for all liver disease discharges increased from 10.4 % in 2000 to 13.1 % in 2008.

**Table 5:** Mortality rate for liver diseases discharges (2000-2008)

Year	Mortality rate by disease category (%)								
	Viral hepatitis	HBV	HCV	Cirrhosis	Alcoholic cirrhosis	Nonalcoholic cirrhosis	HCC	Other^a^	All
2000	0.9	0.9	0.4	13.1	13.4	11.6	26.5	7.8	10.4
2001	1.5	2.3	0.6	14.5	14.4	14.7	26.1	9.4	12.0
2002	1.2	2.0	1.0	14.2	14.7	11.9	24.5	8.7	11.2
2003	1.5	3.3	0.8	13.6	13.4	15.2	28.3	9.3	11.5
2004	1.2	2.8	0.6	13.4	13.4	13.2	25.1	10.6	11.9
2005	1.7	2.3	1.5	13.7	13.7	13.7	26.7	9.5	12.0
2006	1.4	1.5	1.2	14.3	14.4	13.7	25.4	10.4	12.9
2007	2.2	2.1	2.5	15.3	15.1	16.2	24.1	10.8	13.5
2008	2.8	4.8	2.1	14.6	14.9	13.4	22.6	10.4	13.1

^a^Includes: hepatoptosis; alcoholic liver damage unspecified; autoimmune hepatitis; toxic hepatitis; hepatitis unspecified

### Direct costs of hospital admission

**Fig. 3:**
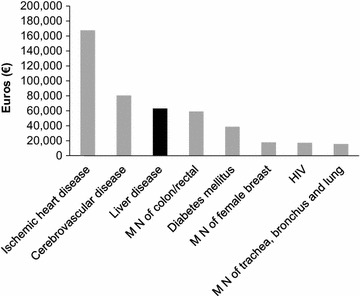
Direct costs incurred during hospital admissions in 2008

Figure 3 shows the direct hospital costs for in-patients for eight disease categories. In 2008, liver disease was associated with the third highest amount payed by the Public Health Service in terms of hospital admissions (€62,950,631), exceeded by ischemic heart disease (€167,538,693) and cerebrovascular disease (€80,387,569).

Alcoholic cirrhosis emerged as the main cost-driver (Table 6), accounting for €26,818,930, 42.6 % of the total cost imposed by liver disease. This proportion far exceeded the cost imposed by HCC, which was €9,737,184 (15.5 %), the second most resource-intensive condition. Table 7 shows the direct costs incurred by hospitals based on an analysis of 148 liver transplants performed during 2008 excluding those associated with follow-up visits and transplant-related therapy such as immunosuppressive drugs. Again, alcoholic cirrhosis and HCC emerged as the main cost-drivers, accounting for €5,876,883 (38.5 %) and € 4,330,335 (28.5 %) of overall costs associated with liver transplants respectively.

**Table 6:** Direct costs incurred during hospital admissions for liver diseases in 2008

Disease category	Disease subcategory	Direct costs (€)	% of direct costs
Viral hepatitis	All	2,647,742	4.2
	HBV	820,639	1.3
	HCV	1,515,620	2.4
Cirrhosis	All	32,138,385	51.1
	Alcoholic cirrhosis	26,818,930	42.6
	Non-alcoholic cirrhosis	5,319,455	8.5
HCC		9,737,184	15.5
Other^a^		18,427,319	29.3
All		62,950,631	100

^a^Includes: hepatoptosis; alcoholic liver damage unspecified; autoimmune hepatitis; toxic hepatitis; hepatitis unspecified

**Table 7:** Costs per hospital admission related to liver transplant in 2008

Disease category	Disease subcategory	Costs (€) relating to liver transplant	% of liver transplant costs
Viral hepatitis	All	618,619	4.1
	HBV	206,206	1.4
	HCV	309,310	2.0
Cirrhosis	All	7,732,741	50.7
	Alcoholic cirrhosis	5,876,883	38.5
	Non-alcoholic cirrhosis	1,855,858	12.2
HCC		4,330,335	28.5
Other^a^		2,577,580	16.9
All		15,259,275	100

^a^Includes: hepatoptosis; alcoholic liver damage unspecified; autoimmune hepatitis; toxic hepatitis; hepatitis unspecified

### Mortality and premature death

Overall, chronic hepatic disease (which includes cirrhosis and HCC) was the 10th most common cause of mortality in Portugal in 2011, causing 21.8 deaths per 100,000 inhabitants (Table 8). However, chronic hepatic disease and HCC are even more important causes of premature death (Table 9), ranking third based on PYLL.

**Table 8:** Causes of death in Portugal during 2011

Disease category	Crude rate/100,000 of the population
Cerebrovascular disease	125.5
Ischemic heart disease	66.0
Pneumonia	51.4
Diabetes mellitus	43.1
Malignant neoplasm of prostate	36.1
Malignant neoplasm colon and rectum	35.9
Malignant neoplasm of trachea, bronchus and lung	35.2
Malignant neoplasm of female breast	29.8
Malignant neoplasm of stomach	23.0
Chronic hepatic disease and cirrhosis/HCC	21.8

**Table 9:** Causes of premature death (less than 70 years of age) in Portugal during 2011 based on PYLL

Disease categories	Lost life years
Motor vehicle traffic accidents	19,974
Malignant neoplasm of trachea, bronchus and lung	19,880
Chronic hepatic disease and cirrhosis/HCC	17,586
Cerebrovascular disease	14,125
Ischemic heart disease	14,113
Malignant neoplasm of colon/rectal	11,540
Malignant neoplasm of stomach	10,158

## Discussion

Liver disease is one of the most common chronic diseases in Portugal. Hospital admissions from hepatic disease peak in males aged between 20 and 60 years. This group is the most socioeconomically active group in terms of contribution to the general economy and to individual families in Portuguese society [18, 19]. The high rate of liver disease in this group is likely to impose a considerable indirect burden on the economy (e.g., through lost productivity, benefit payments and lost taxation) and families.

The pattern of mortality underscores the economic burden imposed by liver disease. Overall, in 2011 liver disease was the tenth most common cause of death in Portugal. However, hepatic disorders were the third most common cause of premature death (i.e., in people less than 70 years of age). Therefore, interventions aimed at reducing the number of cases of liver disease in this group could reduce the socioeconomic burden and mortality imposed by liver disease [20].

This analysis focused on the direct medical costs paid by public health insurance due to hospitalization, medication and human resources, estimated to be €62,950,631 in 2008. Alcoholic cirrhosis was the leading cause of liver-related hospital admission and the main cost-driver, accounting for €26,818,930. Reducing alcoholic cirrhosis through education and legislation could decrease direct costs related to hospitalization and liver transplantation. The probable reduction in other alcohol-related public health problems, as traffic accidents, suggests that our analysis underestimates the potential economic, clinical and societal impact of tackling alcohol abuse [9, 21, 22]. There is evidence supporting the effectiveness and cost-effectiveness of policies to reduce the impact of alcohol consumption. A thorough 2009 review concluded that initiating policies that regulate the environment in which alcohol is marketed are most effective [23]. In particular, measures to make alcohol more expensive, limiting its availability and regulating advertising practices were most cost effective [23]. A more recent report suggested that different interventions are more suitable depending on the prevalence of hazardous alcohol use, with taxation and population-wide interventions most cost-effective in settings of high prevalence and targeted interventions cost-effective for settings of low prevalence [24]. A report as part of the 2005–2007 ELSA (enforcement of national laws and self-regulation on advertising and marketing of alcohol) project found that Portugal had relatively few regulations regarding alcohol marketing compared with other European countries and in fact had no procedures to monitor these regulations [25]. It should be noted that new laws came into force in 2013 in Portugal that increased the legal age for the purchasing of spirits to 18.

Chronic hepatitis B virus (HBV) and HCV are, according to the International Agency for Research on Cancer [26], carcinogens in humans. Between 2 and 5 % of patients with liver cirrhosis due to HCV develop HCC annually [27] and HBV causes approximately 20 % of HCC cases in the western world [28]. In Portugal, the number of discharges due to viral hepatitis almost halved between 2000 and 2008, whereas the number of HCC cases doubled during the same time.

Universal HBV vaccination, introduced in Portugal in 2000, and improved detection as well as effective and well-tolerated treatments for viral hepatitis generally, and HBV and HCV in particular, probably contributed to this reduction [29, 30].

HBV and HCV that are refractory to treatment can progress to decompensated cirrhosis and HCC, resulting in high economic costs for public health services [31]. Worldwide, liver cancer is the sixth most common malignancy (749,000 new cases a year), the third most common cause of cancer-related mortality (692,000 deaths a year) and accounts for 7 % of all cancers [32]. In Western countries, 90 % of people who develop HCC have cirrhosis. Viral infection, alcohol, metabolic syndrome and immune-mediated conditions (such as primary biliary cirrhosis and autoimmune hepatitis) are major risk factors [33]. In Portugal, HCC imposes a considerable economic burden [7]. The socioeconomic burden is even higher when liver transplants and premature deaths are included. However, new anti-viral therapies reduce long-term liver complications and, therefore, result in fewer hospitalizations and less morbidity [34–38]. Future studies should assess the impact of these and other new treatments on the morbidity, mortality and socioeconomic burden imposed by viral liver diseases.

Although studies from several countries suggest that liver disease imposes a considerable socioeconomic burden [1–4, 11], no studies quantify costs imposed by liver disease in Portugal. This study aimed to provide basic data to inform the prioritization of health services. Our results show that interventions to prevent morbidity, mortality and premature death are urgently required. Tackling risk factors—such as intravenous drug use and alcohol consumption, unhealthy nutrition and a sedentary lifestyle—should be the main goal to reduce the societal, clinical and social toll imposed by liver diseases and other chronic non-communicable diseases in Portugal. Our results confirm, for example, that reducing alcohol consumption should be a main target of public health programmes in Portugal [21, 39]. Against this background, managing end-stage liver disease through outpatient clinics that focus on compliance and education improve patient outcomes [40]. These strategies could minimize the direct and indirect costs associated with liver disease, particularly premature morbidity and mortality.

Our study is subject to certain limitations. Firstly, the study does not capture all direct costs from the perspective of the Portuguese National Health Service. For example, the analysis excludes direct costs imposed by ambulatory outpatients, including medication, medical consultations, treatments which do not need hospitalization and other direct costs incurred by visiting medical institutions. Secondly, the study did not estimate the indirect costs arising from, for example, lost workdays and the costs paid by caregivers to support the population with liver disease. However, these limitations mean that the study is likely to underestimate the costs imposed by liver disease to the Portuguese National Health Service and the wider economy. In addition, the PYLL index has its limitations, for example, it underestimates the importance of diseases that contribute to, but are not recorded as, the underlying cause of death, and there is a persistent controversy regarding the definition of an upper end point during its calculation.

## Conclusions

In 2011, liver disease was the 10th most common cause of death and the third most important cause of premature death in Portugal. Alcohol cirrhosis was the leading cause of liver-related hospital admissions between 2001 and 2008. It appears that liver disease represents a considerable social and economic problem in Portugal. Our results suggest that educational, legislative and therapeutic interventions to prevent morbidity, mortality and premature death from liver disease are urgently required.

## Abbreviations

ALOS: average length of stay
DGS: Direcção Geral de Saude
HBV: hepatitis B
HCV: hepatitis C virus
HCC: hepatocellular carcinoma
ICD: international classification of diseases
INE: Instituto Nacional de Estatística
PYLL: potential years of life lost
